# Artemisinin Protects Human Retinal Pigmented Epithelial Cells Against Hydrogen Peroxide-induced Oxidative Damage by Enhancing the Activation of AMP-active Protein Kinase

**DOI:** 10.7150/ijbs.30536

**Published:** 2019-07-25

**Authors:** Shuai Li, Shubhash Chandra chaudhary, Xia Zhao, Uma Gaur, Jiankang Fang, Fengxia Yan, Wenhua Zheng

**Affiliations:** 1 Center of Reproduction, Development & Aging, Faculty of Health Sciences, University of Macau, Taipa, Macau SAR, China; 2 Institute of Translation Medicine, Faculty of Health Sciences, University of Macau, Taipa, Macau SAR, China

**Keywords:** artemisinin, retinal pigment epithelial cells, oxidative stress, AMPK

## Abstract

Dry age-related macular degeneration (AMD), a leading cause of blindness in aged population, is directly associated with oxidative stress induced damage of the retinal pigmented epithelial (RPE) cells. In the current study, we investigated the role of AMPK in the protective effect of artemisinin, an FDA approved anti-malarial Chinese herbal drug, on RPE cell line D407, against H_2_O_2_ induced oxidative stress. Our results showed that artemisinin promoted the survival of D407 cells from H_2_O_2_. Artemisinin reduced intracellular ROS generation and oxidative stress, decreased LDH release and the loss of mitochondrial membrane potential in D407 cells treated with H_2_O_2_. Western blotting showed that artemisinin concentration- and time-dependently stimulated the phosphorylation of AMP-activated protein kinase (AMPK) in D407 cells while AMPK inhibitor Compound C or knock-down of AMPK by si-RNA, inhibited the survival protective effect of artemisinin. More importantly, artemisinin produced a similar protective effect in primary cultured retinal pigment cells which was also blocked by inhibitors of AMPK. Taken together, these results suggested that artemisinin promotes survival of human retinal pigment cells against H_2_O_2_-induced cell death at least in part through enhancing the activation of AMPK. Therefore, artemisinin may be a beneficial therapeutic candidate for the treatment of age-related diseases, including retinal disorders like AMD.

## Introduction

Age-related macular degeneration (AMD) is a chronic age-related degenerative eye disorder^1^, ^2^. which is one of the major causes of blindness globally and more common in developed countries^3^, ^4^. A number of life style factors, including smoking, alcohol consumption and dietary intake, are found to be directly associated with a higher prevalence of AMD^5^, ^6^. Older people mainly suffer from two distinct types of macular degenerations, dry or atrophic, macular degeneration (also called non-neovascular macular degeneration) with drusen and wet or exudative, macular degeneration (also called neovascular macular degeneration). Dry form of macular degeneration is the most common form of AMD where visual loss is usually gradual and irreversible. About 10-15 percent of AMD patients are suffering from the wet form of AMD which is commonly characterized by the development of new blood vessels^7^, known as neovascularization. Wet form of AMD can badly damage the central eye compared to dry form which causes permanent scars in the retina, as a result, patients with wet AMD rapidly lose their visual tendency. Abnormal level of reactive oxygen species (ROS) in the retinal pigment epithelium (RPE) has been found to be associated with the development of AMD^8^. Vision loss due to AMD severely affects people's day-to-day work and activities like reading, walking, crossing roads, seeing and recognizing objects including driving vehicles that lead to depression, falls and road accidents which are in turn associated with an increased risk of mortality in elderly people^9^-^11^.

The imbalance between the antioxidant defense system and the production of ROS like hydrogen peroxide (H_2_O_2_), hydroxyl radical (∙OH), nitric oxide (NO) leads to oxidative stress in cellular environment. These ROS are short-lived, high energetic species which are produced during metabolic reactions in mitochondria. It has been found that oxidative stress severely causes RPE degeneration and it consequently, plays a crucial role in the development and progression of different kinds of eye disorders including AMD^12^, ^13^. A pathologically large amount of ROS production will exert oxidative stress to the eyes which directly or indirectly affect the cellular functions and morphological changes of RPE and their damage^14^-^16^, and also damage to the DNA and mitochondria^17^. Age-related degeneration of RPE causes the death of photoreceptor cells in the eyes which leads to the loss of central vision^18^. The number of people living with AMD is expected to reach about 196 million worldwide by 2020 and increase to 288 million by 2040^19^. There are no specific and reliable drugs for AMD treatment except antibody against vascular endothelial growth factor which is commonly used for the treatment of wet AMD. Therefore, a new therapeutic drug is essential to be developed for the better treatment of AMD patients.

Artemisinin, an herbal natural product, extracted from Chinese medicinal plant named *Artemisia annua* or, sweet wormwood, also known as Qinghaosu. Artemisinin and its different derivatives including dihydroartemisinin, artesunate, artemether have been clinically used as anti-malarial and anti-fever^20^-^22^. Moreover, in addition to its strong anti-malarial activity, artemisinin also shows its potent anti-tumor and anti-cancer^23^-^25^, anti-allergic or anti-inflammatory^26^, anti-viral^27^, anti-helminthese and anti-protozoan parasitic^28^, ^29^. The 2015 Nobel Prize winner, Prof. Youyou Tu in Physiology and Medicine has discovered artemisinin and its clinical application for malaria therapy. More recently, we have reported that artemisinin, in clinical relevant dosage, promoted PC12 and cortical neuron cells survival against nitric oxide-induced toxicity and human retinal pigmented cells (D407) from hydrogen peroxide-induced cell damage^30^, ^31^. Clinical uses of artemisinin and their derivatives are safe with no major toxic side effects, and are potent and effective in humans that further support its development as a new potential therapeutic candidate against AMD.

It has been reported that AMP-activated protein kinase (AMPK) plays pivotal role not only in regulating cell apoptosis, cellular energy homeostasis but also cell survival under stress conditions^32^-^34^. AMPK induction is required to carry out many vital cellular functions such as cytoprotection. Various cellular conditions like serum starvation, lack of oxygen content and glucose deprivation are essential for the activation of AMPK^8^, ^35^, ^36^. Our previous reports have shown that artemisinin exerts protective effects on D407 cells, a human RPE cell line, against hydrogen peroxide^30^, ^31^, but the underlying molecular mechanisms is still need to be elucidated and the role of AMPK on the protective effect of artemisinin is still not known.

In the current study, we applied a model of oxidative stress by using a well-known oxidant, hydrogen peroxide (H_2_O_2_) that produce ROS during cellular metabolism in human RPE cell line D407 cells and human primary cultured RPE cells. We investigated whether there is any involvement of AMPK and its activation is implicated in cell survival. We demonstrated that upon the activation of AMPK by artemisinin stimulation, D407 cells were protected from H_2_O_2_ toxicity while AMPK inhibitor compound C or the decreased expression of AMPKα with si-RNAs targeting AMPKα, significantly abolished the protective effects of artemisinin. Moreover, artemisinin has similar effect on human primary cultured RPE cells. Taken together, these results thus give a key mechanistic support suggesting that artemisinin promotes survival of human RPE cells against H_2_O_2_-induced cell death at least in part through activation of AMPK.

## Materials and Methods

### Materials

Dulbecco's Modified Eagle's Medium (DMEM), Fetal Bovine Serum (FBS), Bovine Serum Albumin (BSA) and Trypsin (0.5% EDTA) were obtained from GIBCO^TM^, (Invitrogen Corporation). Artemisinin, Penicillin/Streptomycin, Lipofectamine^R^ 2000 reagent (Invitrogen Co.,CA, USA), DMSO, H_2_O_2_ were obtained from Sigma Aldrich (St. Louis, MO, USA). Sodium Azide (NaN_3_) were obtained from Acros Organic, (New Jersey, USA), and 3-(4,5-dimethylthiazol-2-yl)-2,5-diphenyl tetrazolium bromide (MTT), Cell ROXs Deep Red Reagent, and Hoechst 33342 were purchased from Molecular Probes (Eugene, or, USA). Pierce BCA protein assay kit and Halt^TM^ Protease and phosphatase inhibitor cocktail were purchased from Thermo Scientific USA, and 5,5′,6,6′-tetrachloro-1,1′,3,3′-tetraethyl-benzimidazolyl-carbocyanineiodide (JC-1) from Beyotime, Annexin V-FITC/PI were purchased from BBI Life Sciences. anti-p-AMPK, anti-AMPK and anti-β-Actin antibodies were purchased from Cell Signaling Technology (CST) (Beverly, MA, USA). Anti-Rabbit IgG HRP-conjugated secondary antibody was purchased from Promega (Madison, Wl, USA).

### Cell Culture and Transfection

Human retinal pigment epithelial cell line (D407) was obtained from Cell bank, Sun Yat-Sen University (Gauangzhou, China). Cells were grown in DMEM Dulbecco's Modified Eagle's Medium) supplemented with 10% fetal bovine serum (FBS) and 100 μg/ml streptomycin, 100 units/ ml of penicillin and kept at 37°C under humidified atmosphere with 5% CO_2_. Cells were transiently transfected either with sh- or si-RNA using Lipofectamine 2000 (Invitrogen) per the manufacturer's instructions. All transfections were carried out for 48 hours.

Primary RPE Cell Culture: Primary RPE cells were prepared as we described 37. In brief, the anterior half of the eye was separated from the posterior half of the eye. The retina was gently removed from the posterior portion, followed by treatment with trypsin 0.25% and EDTA 0.02% to release RPE cells from the posterior half of eye. To purify RPE cells, the isolated cells were suspended in Percol 40% with 0.01 mol/L Na_2_PO_4_ and 0.15 mol/L NaCl (pH 7.4) and followed by a density gradient centrifugation. After centrifugation, pellets were collected and RPE cells were cultured in Miller medium.

### MTT Assay

The cell viability of D407 cells was measured by MTT [3-(4,5-dimethylthiazol-2-yl)-2,5-diphenyltetrazolium bromide] cell viability assay. D407 cells were seeded in 96-well plates (5Χ10^3^ cells/well) either with 1% FBS or non-serum containing medium. Serum starved cells were treated with artemisinin in different concentration (3.125 μM to 100 μΜ) for 2 h. After 2 h, media was removed and cells were exposed to 100 μM H_2_O_2_ for 24 h. For cytotoxic effect of H_2_O_2,_ cells were exposed to H_2_O_2_ (3 to 1000 μM) for 24 h. For AMPK inhibitor (Compound C), 5 μΜ AMPK inhibitor was addd for 30 min before artemisinin treatment. After 2 h, media was removed and cells were exposed to 100 μM H_2_O_2_. After 24 h, MTT (0.5 mg/ml) was added into each well and further incubated at 37°C for 3-4 h till the formation of formazan crystals. The medium containing MTT was then removed from each well, and 100 μl DMSO solution was added to each well to dissolve the crystals. The absorbance of each well was recorded at 570 nm using a BIO-RAD680 micro-plate reader (Thermo, Walsam, MA, USA). Cell viability was then calculated as a percentage compared with the control group.

### Hoechst 33342 staining

D407 cells seeded on 96-well plates pretreated or not pretreated with 10 μM artemisinin for 2 h were incubated with 100 μM H_2_O_2_ for 24h at 37°C , followed by washing with 1XPBS and then fixed with 4 % paraformaldehyde for 10~15 min on ice. The fixed cells were then washed and stained with Hoechst 33342 (10 μg/ml) in PBS for 5-10 min at room temperature. After washing gently twice, cells images were taken with high content screening system (ArrayScanVTI, Thermo Fisher Scientific, USA) at excitation 343 nm and the emission 483 nm wave length. The apoptotic counts of cells were observed, counted and their percentage were analyzed.

### Measurement of Mitochondrial membrane potential (△ψm)

JC-1 staining dye was used to measure the changes in mitochondrial membrane potential and metabolic activities of the cells. Briefly, D407 cells were seeded into 96-well plates (1Χ10^4^ cells/well). And treatment with 10 μM artemisinin for 2 h. After 2 h, media was removed and cells were exposed to 100 μM H_2_O_2_ for 24 h, cells were incubated with JC-1 dye (10 mg/l) in fresh medium for 30 min at 37°C as per the manufacturer's instructions. After incubation for appropriate time, cells were washed twice with ice-cold PBS. For the fluorescence signal, the intensities of red fluorescence (excitation 560 nm, emission 595 nm) and green fluorescence (excitation 485 nm, emission 535 nm) were captured by infinite M200 PRO multimode microplate reader fluorescence microscope. Mitochondrial membrane potential (△ψm) was measured as the ratio of red /green fluorescence intensities and was normalized with the control group.

### Measurement of reactive oxygen species (ROS)

Intracellular reactive oxygen species (ROS) generation was evaluated by Cell ROXs Deep Red Reagent (Thermo Fisher Scientific, USA). Briefly, the cells grown in 96-well plates and treatment with 10 μM artemisinin for 2 h. After 2 h, media was removed and cells were exposed to 100 μM H_2_O_2_ for 24 h. Cells were then incubated with Cell ROXs Deep Red Reagent (5μM) in fresh DMEM for 1h in dark. Cells were washed with 1X PBS. The fluorescence was measured by fluorescence microscope with a high content screening system (ArrayScanVTI, Thermo FisherScientific, USA) at excitation 640 nm and the emission 665 nm wave length. ROS level was semi-quantified and percentage of ROS level was normalized with the control group.

### LDH Measurement

Cell cytotoxicity was also evaluated by measuring the activity of lactate dehydrogenase (LDH) released into the cultured medium. Briefly, D407 cells were seeded into 96-well plates (1Χ10^4^ cells/well). After the treatment with drugs, the activity of LDH released in the medium was determined according to the instructions of Cyto Tox-ONE™ Homogeneous Membrane Integrity Assay (Promega, USA). The fluorescent intensity was measured using Infinite M200 PRO Multimode Microplate at an excitation of 560 nm and emission at 590 nm wave length. The values LDH released were normalized as percentage to the control group.

### Western Blotting

Cells either treated or non-treated with drugs were harvested, washed with cold phosphate-buffered saline (PBS) and lysed in radio immunoprecipitation assay (RIPA) buffer (50 mM Tris-HCl, pH 7.4, 1% NP40, 0.1%SDS, 150 mM NaCl, 5mM EDTA) containing freshly added protease and phosphatase inhibitors cocktail purchased from Thermo Scientific, USA or cells were lysed with 2X sample buffer [ 62.5 mM Tris-HCl pH (6.8), 2 % (w/v) SDS, 10 % glycerol, 50 mM dithiothreitol, 0.1 % (w/v) bromophenol blue]. Lysed cells were centrifuged at 13,000 g for 15 min, lysate was used for protein quantification using BCA protein assay kit (Thermo scientific), according to the manufacturer's instructions. Proteins were resolved by SDS-PAGE (polyacrylamide gel electrophoresis) and transferred to a nitrocellulose membrane (Whatman^R^, PROTRAN^R^). Membranes were blocked in 1% BSA in PBST (BSA/PBS/1% Tween) for 1 hour. Primary antibodies at a 1:1000 dilutions were added in 1% BSA in 1X PBST and incubated overnight at 4^0^ C. Membranes were washed three times with 1X PBST and then incubated with horseradish peroxidase conjugated anti-rabbit secondary antibody (Invitrogen) at a dilution of 1:5000 for 1 hour at room temperature and immunoblotting was performed using ECL detection kit reagent (BIO-RAD, Clarity™ Western ECL Substrate, 200 ml #1705060).

### Flow Cytometry Assay

D407 cell (5 × 10^5^ per well) were seeded into 6-well plates. And treatment with 10 μM artemisinin for 2 h. After 2 h, media was removed and cells were exposed to 100 μM H_2_O_2_ for 24 h. Then, cells were harvested and washed two times with ice-cold PBS and suspended in Annexin V binding buffer. The cell supernatant was stained with 5 μL of Annexin-V-FITC and 10 μL of propidium iodide (PI) solution. The number of apoptotic cells was analyzed using the flow cytometry (BD Accuri®C6). Experiment was repeated three times.

### Statistical analysis

All the data were presented as mean ± SEM. Each experiment was performed in triplicates. Statistical differences were analyzed by one-way ANOVA (Analysis of variance) in combination with a post-hoc test, and p values <0.05 were regarded as statistically significant.

## Results

### Artemisinin protected human retinal pigmented (D407) cells from H_2_O_2_-induced cell toxicity

**Fig 1:**
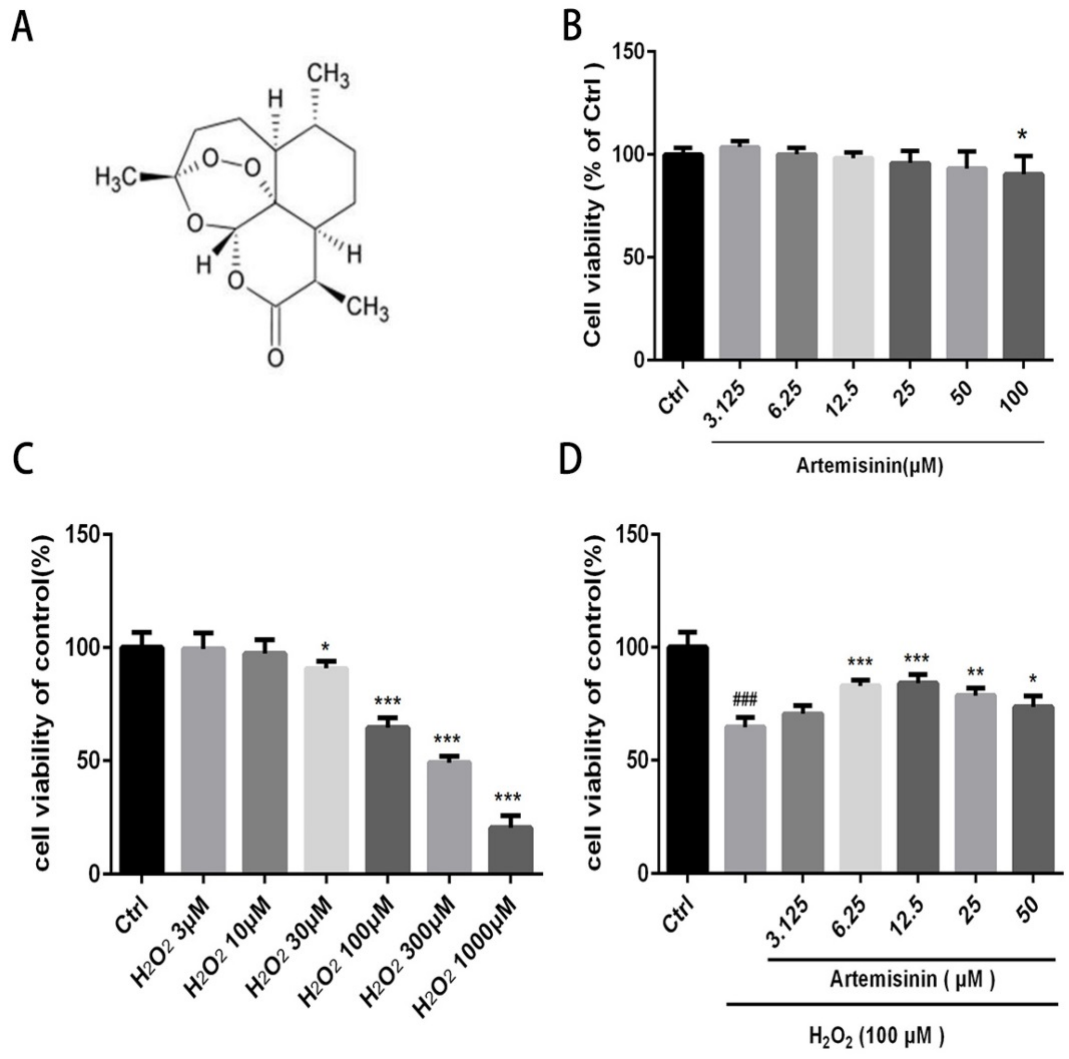
** Artemisinin increased cell viability in D407 cells treated with H_2_O_2_.** (A) Chemical structure of Artemisinin. (B) Cytotoxicity of artemisinin in D407 cells in dose-dependent manner. D407 cells grown in 96-well plate were treated with different concentrations of artemisinin for 24 h, and cell viability was analyzed by MTT assay. *p<0.05 verses control group. (C) Cytotoxicity of H_2_O_2_ in D407 cells in dose-dependent manner. D407 cells grown in 96-well plate were treated with different concentrations of H_2_O_2_ for 24 h, and cell viability was analyzed by MTT assay. *p<0.05 verses control, ***p<0.001 verses control group. (D) Protective effects of artemisinin on H_2_O_2_-induced cell death as determined by MTT cell viability assay; D407 cells pre-treated with different doses of artemisinin as indicated for 2 h, were exposed to 100 μM H_2_O_2_ for 24 h and absorbance was taken at 562 nm by micro-plate reader for cell viability analysis. Results are shown as the mean ± SEM. Three independent experiments were performed with similar results. ###*p<0.001* versus control group, *p<0.05versus H_2_O_2_- treated group, **p<0.005 versus H_2_O_2_- treated group. ***p<0.001 versus H_2_O_2_- treated group.

We first investigated the cytotoxic effect of artemisinin and H_2_O_2_ on D407 cells. The cells were incubated with increasing concentration of artemisinin and H_2_O_2_ in 96-well plates for 24 h. After 24h, the cell viability was measured by MTT assay. As shown in (Fig. 1B and 1C), artemisinin did not cause any cytotoxicity in D407 cells from 3.125 μM to 50 μM, and H_2_O_2_ induced cell death in a concentration-dependent manner and it reached maximum at 1000 μM of H_2_O_2_. Approximately 35-40% of the cells died when exposed to 100 μM H_2_O_2_ for 24 h and this concentration was chosen for further experiments. We then, analyzed whether artemisinin protected the cells against H_2_O_2_-induced cell death. Cells pre-treated with artemisinin in various concentrations as indicated for 2 h in 96-well plates were further incubated with or without H_2_O_2_ for 24 h_._ Cell viability was then measured by MTT assay. We observed that artemisinin protected the cells from H_2_O_2_ induced cell damage in dose-dependent manner (Fig. 1D). Artemisinin showed its protective effect even at very low concentration i.e. 6.25 μΜ. and reached maximum at 12.5 μM concentration of artemisinin. These results suggested that artemisinin promoted D407 cell survival from H_2_O_2_-induced cell death.

### Artemisinin prevented apoptosis of D407 cells mediated by H_2_O_2_

**Fig 2:**
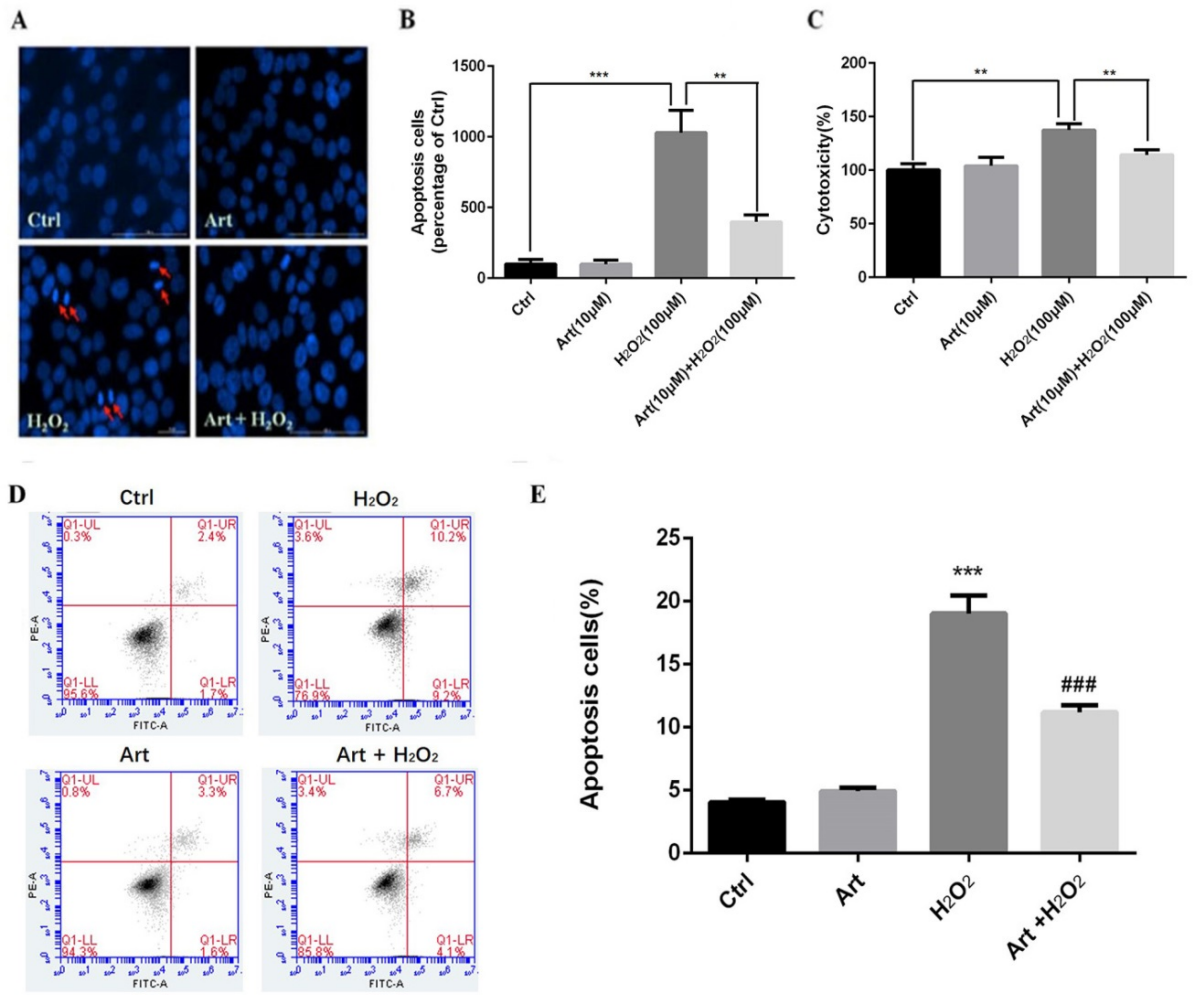
** Protective effect of artemisinin on H_2_O_2_-induced D407 cells apoptosis.** (A)D407 cells pre-treated with artemisinin (10 μM) for 2 h were exposed with or without H_2_O_2_ (100 μM) for 24 h. Apoptotic cells were observed by Hoechst 33342 staining as shown in fluorescence images taken with fluorescence microscope. The photographs of apoptotic cells with condensed chromatin are shown with arrow head (B) The graph displaying the percentage of apoptotic cell nuclei (right), n=200~250. Results are shown as the mean ± SEM and represent three independent experiments, ***p<0.0001 versus control group, **p<0.001 verses H_2_O_2_-treated group. (C) D407 cells pre-treated with artemisinin (10 μM) for 2 h were incubated with H_2_O_2_ (100 μM) for 24 h and LDH released was measured. The graph depicting the percentage of cytotoxicity. Results are shown as the mean ± SEM. Three independent experiments were performed in triplicates. ***p<0.0001 versus control and H_2_O_2_-treated groups. (D, E) cells pre-treated with artemisinin (10 μM) for 2 h were exposed with or without H_2_O_2_ (100 μM) for 24 h, and apoptosis of D407 cells was determined by flow cytometry, H_2_O_2_ induced apoptosis, while artemisinin reversed the effects of H_2_O_2_. ***p<0.001 versus control group, ### *p<0.001* verses H_2_O_2_-treated group.

We investigated the protective role of artemisinin on H_2_O_2_-mediated apoptosis. To examine whether there is apoptotic cell death mediated by H_2_O_2_, we stained the H_2_O_2_-treated D407 cells in the presence or absence of artemisinin with Hoechst 33342 staining. Cell nuclei got condensed and cell morphology changed after exposure with 100 μM H_2_O_2_ for 24 h as observed by Cell image analyzer as shown in the figure with arrow head (Fig. 2A). Nevertheless, the condensation of cell nuclei got substantially reduced and cell morphology also changed by the pre-treatment with 10 μM artemisinin for 2 h (Fig. 2A). Additionally, the number of apoptotic cell death induced by H_2_O_2_ is further reduced with the pre-treatment of artemisinin as shown in bar graph (Fig. 2B). The flow cytometer results suggested that artemisinin inhibited the cell apoptosis induced by hydrogen peroxide (Fig 2D and E) and consistent with the result of Hoechst 33342 staining.

### Artemisinin promoted D407 cells survival via attenuating H_2_O_2_-induced LDH release

Treatment with 100 μΜ H_2_O_2_ of D407 cells for 24 h profoundly increased the release of LDH, about 40% higher compared to non-treated cells. Furthermore, the effect of artemisinin on LDH release induced by H_2_O_2_ was examined. It was found that increased of LDH released induced by H_2_O_2_ was significantly reduced about 25-35% by the pre-treatment with 10 μM artemisinin for 2 h (Fig. 2C). These results suggested that artemisinin protected the cells with the decrease of H_2_O_2_-induced LDH release in the extracellular environment.

### Artemisinin reduced H_2_O_2_-induced mitochondrial membrane potential (△ψm) loss

**Fig 3:**
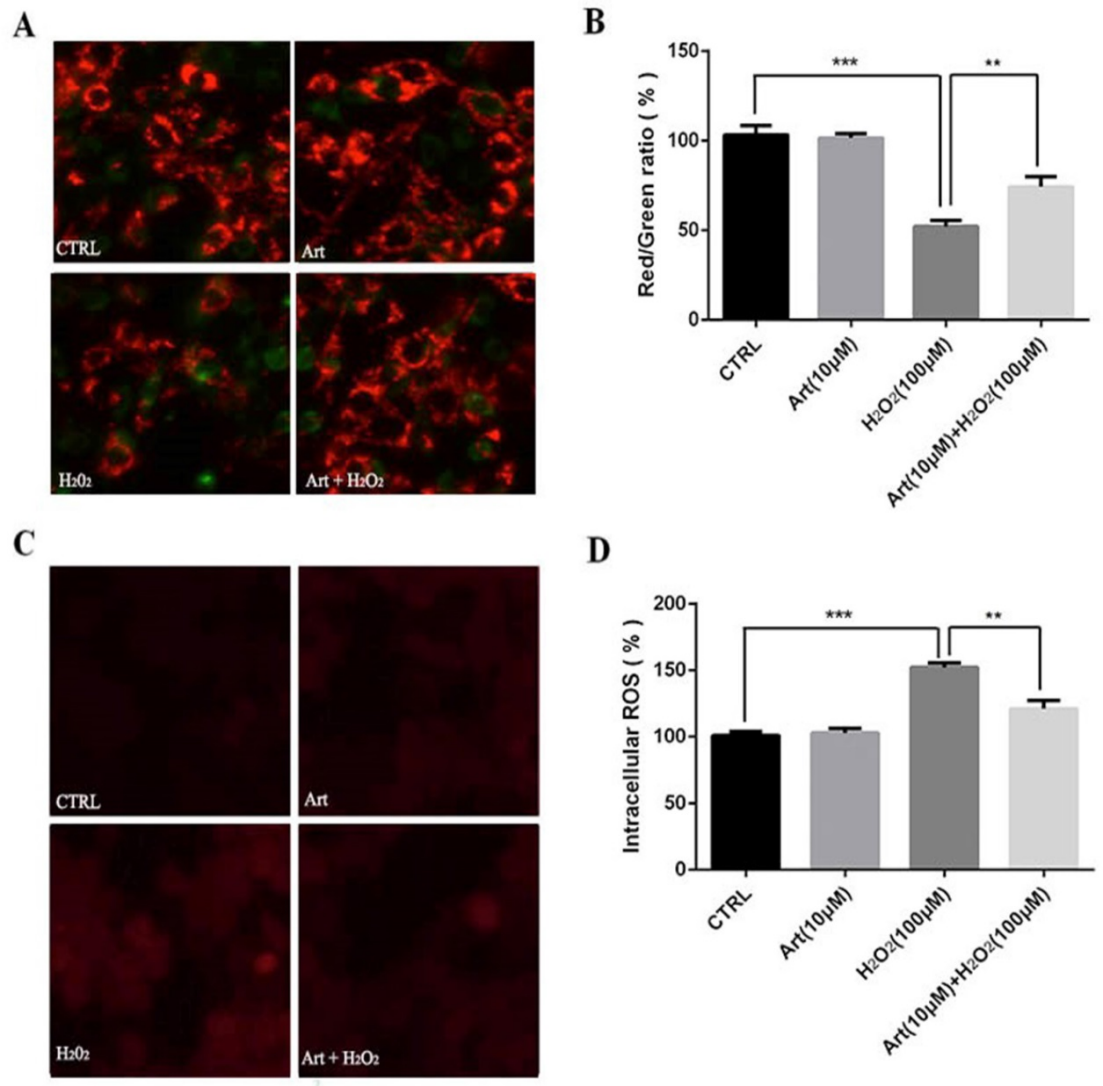
** Artemisinin attenuated mitochondrial membrane potential loss and decreased the production of ROS in D407 cells treated with H_2_O_2_.** (A) D407 cells pre-treated with artemisinin (10 μM) for 2 h were exposed with or without H_2_O_2_ (100 μM) for 24 h. Images shown were taken with fluorescence microscope by staining the cells with JC-1 dyes. Fluorescence intensity of images depicts the change of mitochondrial membrane potential (△ψm). (B) Bar graph displaying the red to green fluorescence intensity ratio represents the loss of mitochondrial membrane potential. Results are shown as the mean ± SEM. Three independent experiments were performed in triplicates. ***p<0.001 versus control group, **p<0.01 versus H_2_O_2_- treated group. (C) D407 cells treated with or without artemisinin (10 μM) for 2 h were further exposed with H_2_O_2_ (100 μM) for 24 h. Images shown were taken with fluorescence microscope. The fluorescence intensity shown in fluorescence images represents the intracellular ROS level as determined by the CellROXs Deep Red Reagent. (D) The bar graph shows the percentage of intracellular ROS level. Results are shown as the mean ± SEM. Three independent experiments were performed in triplicates. ***p<0.01 versus control group ,**p<0.01 versus H_2_O_2_- treated group.

Mitochondrial membrane potential (△ψm) after the exposure of cells with 100 μM H_2_O_2_ for 24 h was measured. We found that the mitochondrial membrane potential (△ψm) was substantially decreased by H_2_O_2_ compared to either non-treated cells or the cells treated with artemisinin alone as shown in fluorescence images. The decrease in red fluorescence intensity and increase in green fluorescence intensity implies the decrease of mitochondrial membrane potential. The decreased mitochondrial membrane potential by H_2_O_2_ was further elevated by pre-treatment with artemisinin as measured by red/green fluorescence intensity ratio of JC-1staining (Fig. 3A and B). These results, thus indicated that artemisinin was able to reverse the decrease of mitochondrial membrane potential induced by H_2_O_2_ treatment.

### Artemisinin reduced H_2_O_2_-induced intracellular ROS generation

Treatment with 100 μM H_2_O_2_ for 24 h increased the production of intracellular ROS in D407 cells about 2 to 2.5 folds compared to non-treated or artemisinin alone treated cells respectively and this increase of ROS production induced by H_2_O_2_ was robustly attenuated by the pre-treatment with artemisinin as shown by figure 3C and D. The observed results are consistent with our previous reports. These results, thus suggested that artemisinin promoted D407 cell survival in H_2_O_2_ - induced cell death by attenuating the intracellular ROS generation.

### Artemisinin stimulated the activation of AMPK in D407 cells

**Figure 4:**
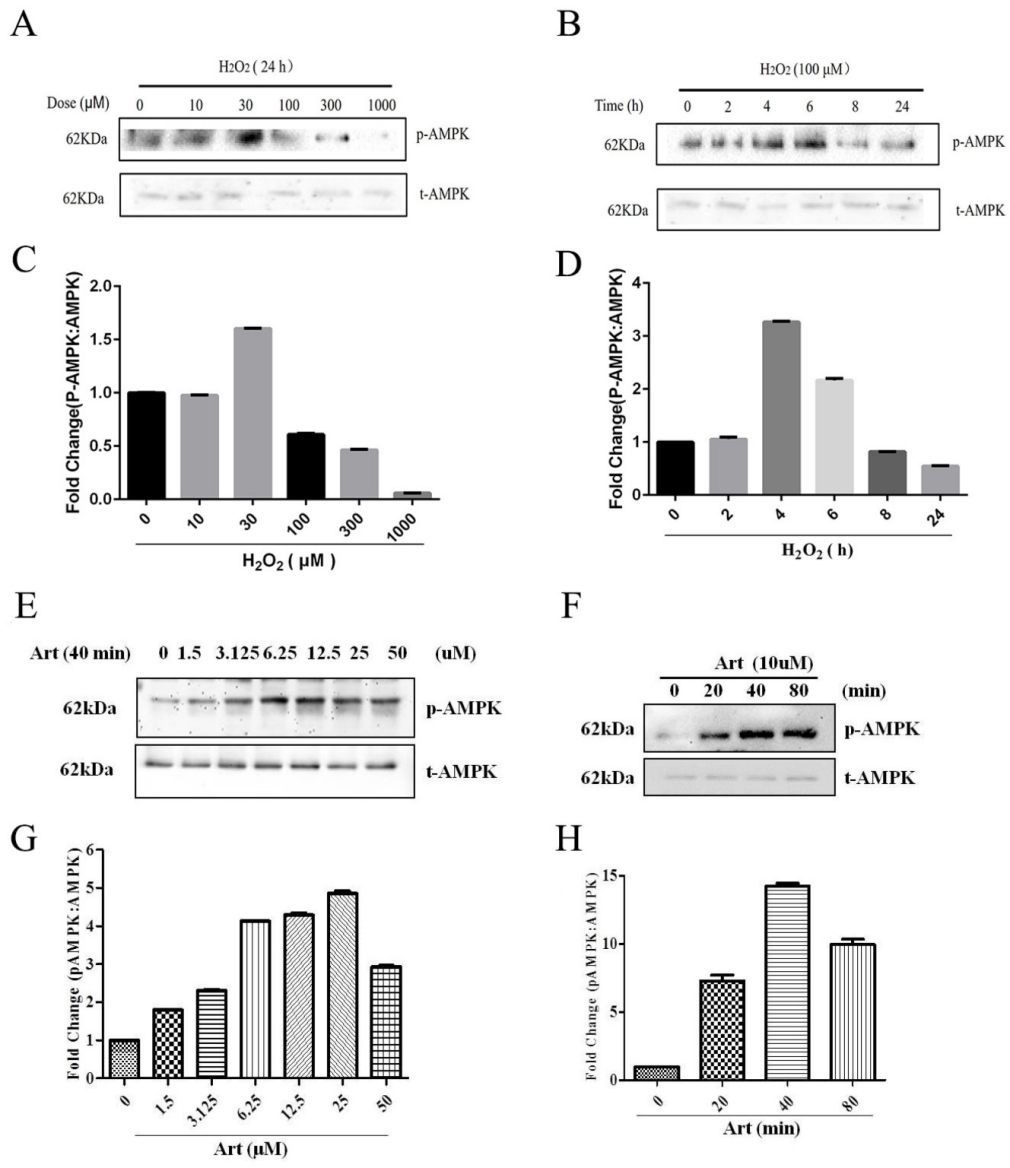
** Involvement of AMPK in cytoprotective effect of artemisinin.** D407 cells treated with H_2_O_2_ at indicated doses for 24 h (A), and with 100 μM H_2_O_2_ at indicated time points (B). Cells were lysed and immunoblotting was performed using anti-pAMPK, anti-AMPK respectively. Results show the expression level of indicated proteins in dose and time-dependent manner. (C) and (D).Then, D407 cells treated with artemisinin at indicated doses for 40 min (E), and with 10 μM artemisinin at indicated time points (F). Cells were lysed and immunoblotting was performed using anti-pAMPK, anti-AMPK respectively. Results show the expression level of indicated proteins in dose and time-dependent manner. (G) and (H), the densitometry graphs represents the quantification of respective protein bands (p-AMPK and total-AMPK ratio).

As shown in Western Blotting, pAMPKα was downregulated in 100μΜ H_2_O_2_ treatment for 24h (Fig. 4 A, B) and artemisinin upregulated in dose-dependent manner of artemisinin treatment as indicated (Fig. 4 E, F). It was found that AMPK was activated with as small as 1.5 μΜ concentration of artemisinin and phosphorylation became maximum at 25 μΜ concentration of artemisinin (Fig. 4 E and G). We then, chose 10 μΜ ART to do time cause study as this concentration stimulating a significant and almost maximal phosphorylation of AMPK in concentration experiment (Figure 4E and G). Our results showed that 10 μΜ ART time-dependently stimulated the phosphorylation of AMPK (Figure 4F and H). These results indicated that artemisinin might protect the D407 cells from H_2_O_2_ toxicity by activating AMPK.

### AMPK was involved in the protective effect of artemisinin against H_2_O_2_ induced toxicity

**Figure 5:**
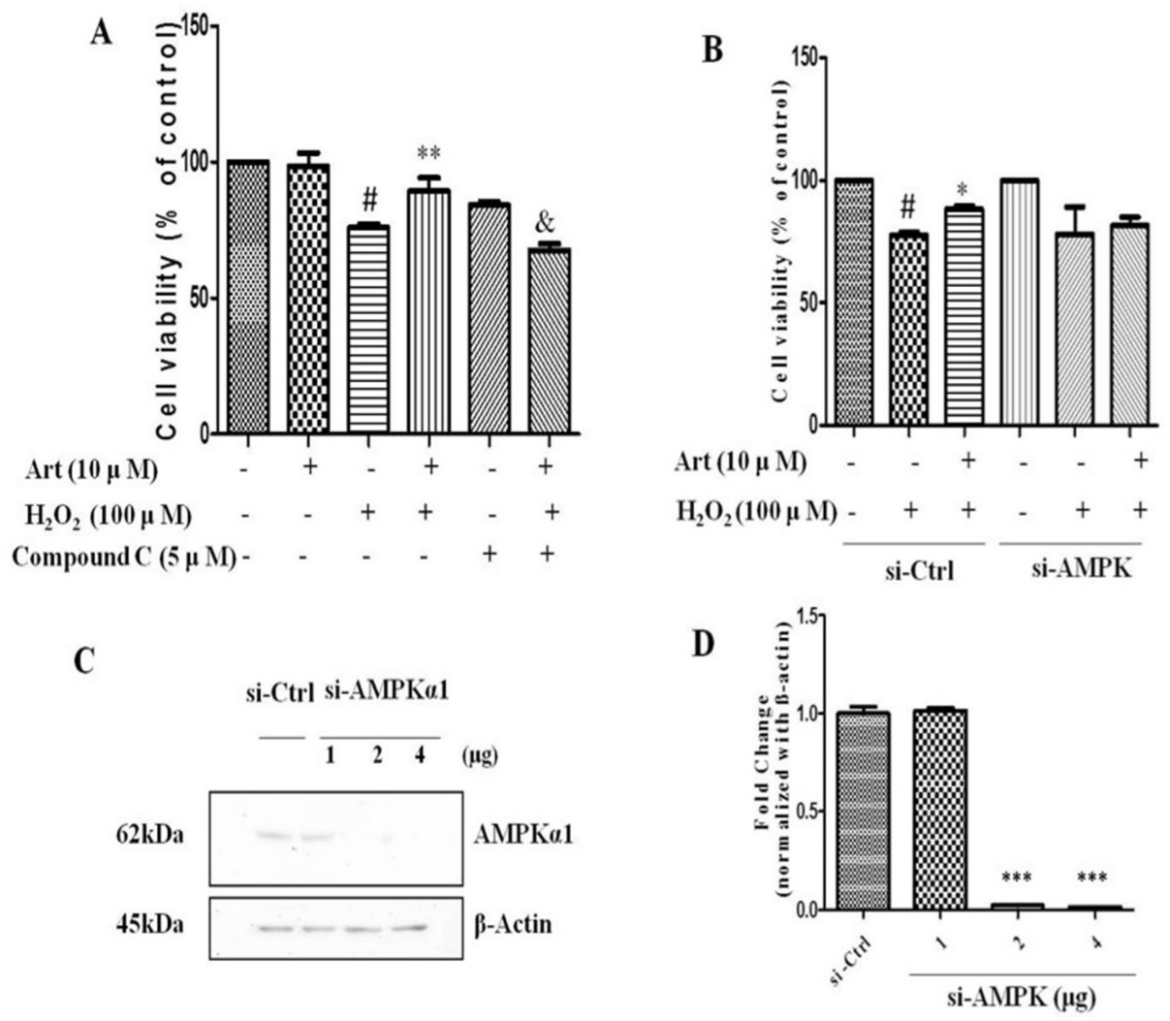
** Inhibition or silencing of AMPKα1 attenuated the protective effect of artemisinin against H_2_O_2_ toxicity in D407 cells.** D407 cells pre-treated either with AMPK inhibitor Compound C as indicated dose (A) or transfected with si-CTRL and/or si-AMPKα (B) , then cells treated with artemisinin (10 μM) for 2 h were exposed with or without H_2_O_2_ (100 μM) for 24 h in 96-well plate. Cell viability was measured by MTT assay. Representative graphs displaying the percentage of cell viability. Results are shown as the mean ± SEM. Three independent experiments were performed in triplicates. ^#^p<0.05, versus control group; ^*^p<0.01,^**^p<0.01, versus H_2_O_2_- treated group; ^&^*p*<0.05, versus H_2_O_2_+ artemisinin group. (C) Immunoblotting with anti-AMPKα1 showing the knock-down efficiency of AMPKα1 with indicated doses of si-AMPKα1. β-actin used as a loading control. (D) The densitometry graphs representing the quantification of protein bands (p-AMPK and β-actin ratio) (right). ^***^p<0.0001, versus si-control group.

**Figure 6:**
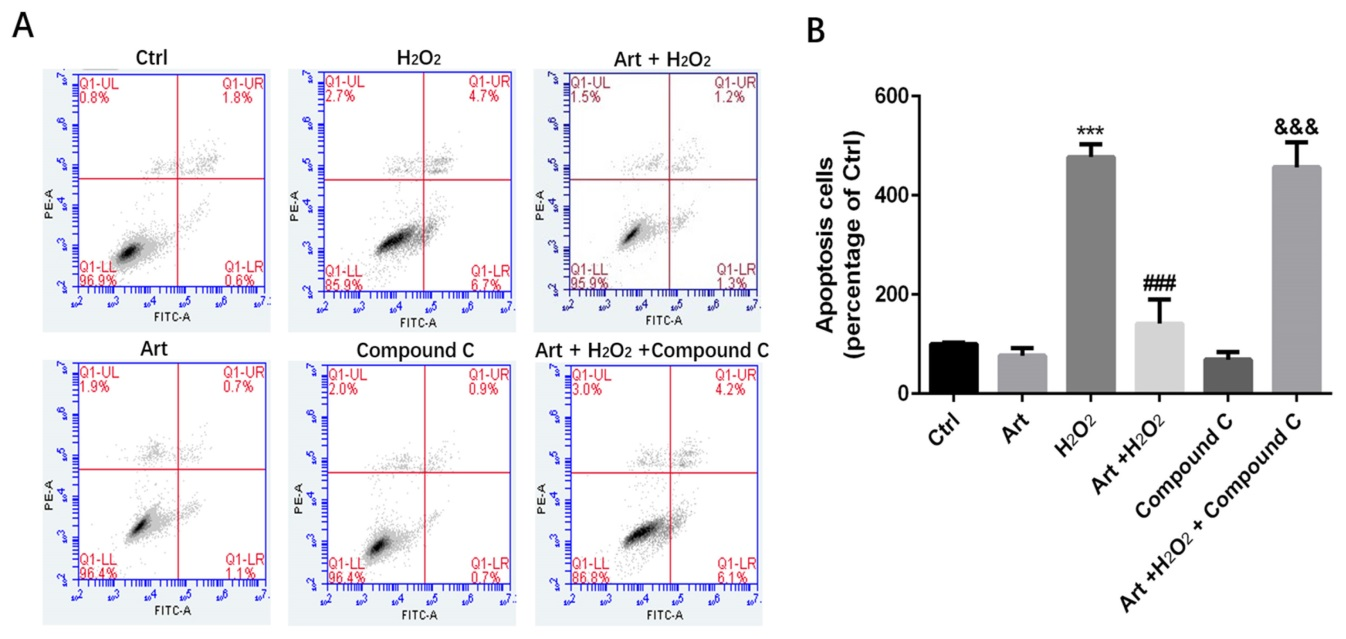
** Protective effects of artemisinin towards cell injury induced by H_2_O_2_ can be reversed by AMPK inhibitor Compound C.** D407 cells pre-treated with AMPK inhibitor Compound C as indicated dose(5μM). Then cells treated with artemisinin (10 μM) for 2 h were exposed with or without H_2_O_2_ (100 μM) for 24 h, and apoptosis of D407 cells was determined by flow cytometry. H_2_O_2_ induced cell apoptosis while artemisinin reversed the effects of H_2_O_2._ After pre-treated with AMPK inhibitor Compound C, the protective effect of artemisinin was reversed. ***p<0.001, verses control group,^ ###^*p<0.001* verses H_2_O_2_-treated group,^ &&&^ *p*<0.001 verses H_2_O_2_+ artemisinin group.

Next, we examined the role of AMPK in protective effect of artemisinin in H_2_O_2_-induced cell apoptosis. To investigate the potential role of AMPK in cell survival, we performed MTT assay for cell viability. To analyze it, biochemical and pharmacological methods were applied. We used AMPK inhibitor, Compound C (5 μM) as described in material and methods. As shown in Fig. 5A, 5 μΜ of Compound C treatment significantly decreased the percentage of live cells compared to Compound C non-treated cells in the presence of H_2_O_2_ in artemisinin pre-treated cells, indicating the role of AMPK in artemisinin cell protection. This hypothesis was also supported by the results in flow cytometer assay (Fig 6). To further validate this finding, we knocked-down AMPKα with si-RNA specific to AMPKα and evaluated the artemisinin protective effect (Fig. 5C and D). Interestingly, knock-down of AMPK robustly eliminated the cell protection tendency of artemisinin in H_2_O_2_-induced cell toxicity (Fig. 5B). It consequently, implies that AMPK plays a pivotal role to promote artemisinin's cell survival effect. Therefore, the results suggested that artemisinin protected the retinal pigmented cells from H_2_O_2_-induced cell apoptosis through AMPK activation.

### Artemisinin produced a similar protective effect in primary cultured retinal pigment cells which was also blocked by inhibitors of AMPK

**Figure 7:**
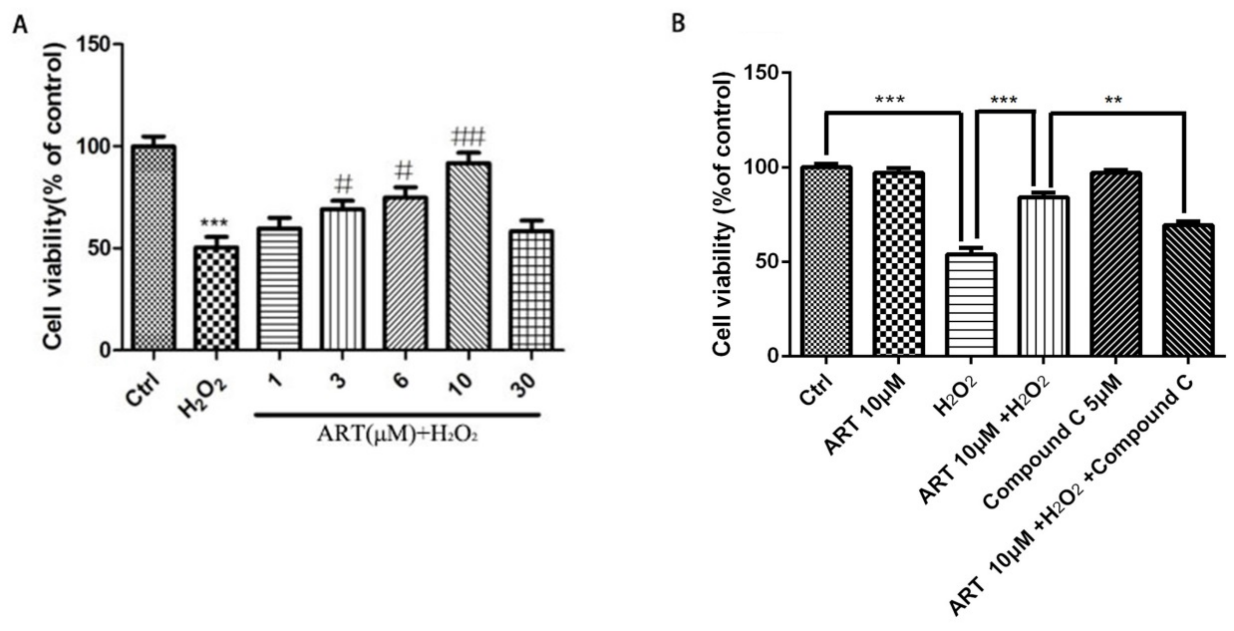
** Protective effects of artemisinin towards cell injury induced by H_2_O_2_ in primary cultured RPE cells.** Primary RPE cells pre-treated with different doses of artemisinin as indicated for 2 h, were exposed to 100 μM H_2_O_2_ for 24 h, and cell viability was analyzed by MTT assay. ***p<0.001, verses control group; ^#^*p*<0.05, ^##^*p*<0.01, versus H_2_O_2_-treated group. (B) Primary RPE cells pre-treated with AMPK inhibitor Compound C(5 μM) for 30min were further incubated with artemisinin (10 μM) for 2 h ,followed by exposure with or without 100 μΜ H_2_O_2_ for 24 h in 96-well plate. Cell viability was measured by MTT assay. **p<0.01, ***p<0.001 versus control , H_2_O_2_-treated groups and H_2_O_2_+ artemisinin group.

All above experiments are performed in cell line D407, to further verify the protective effect of artemisinin in RPE cells, we established the human primary culture RPE cells and studied the protective effect of artemisinin in these cells. Thus, human primary culture RPE cells pre-treated with artemisinin in various concentrations as indicated for 2 h in 96-well plates were further incubated with or without H_2_O_2_ for 24 h. Cell viability was then measured by MTT assay. Similar to the results in D407 cells, Artemisinin concentration-dependently promoted the survival of primary culture RPE cells from H_2_O_2_ injury (Fig 7A). Figure 7B showed that AMPK inhibitor Compound C blocked the protective effect of artemisinin on the human primary culture RPE cells as that of D407 cell.

## Discussion

In recent years, H_2_O_2_ toxicity and its underlying mechanism have been widely studied in both mammalian and bacterial cells. In this study, we provided evidence on the protective activity of artemisinin in D407 cells and primary cultured RPE cells against H_2_O_2_-induced injury is mediated by the activation of AMPK. The results clearly indicate that H_2_O_2_ in a concentration-dependent manner resulted in RPE cells death, while pretreatment with artemisinin protected the cells from H_2_O_2_ -induced oxidative damage, an effect correlated with activation of AMPK.

There are several factors which are directly or indirectly associated with pathogenesis of AMD, among them oxidative stress has been considered as one of the decisive factors that plays a substantial role in progression and development of AMD^38^. Aberrantly high level of ROS is one of the pivotal regulators for the oxidative stress in RPE cells and therefore, for the pathogenesis of AMD. Mitochondria, the major sources of ROS are abundantly found in RPE cells which are implicated in ROS production and in turn oxidative stress. Oxidative stress and ROS are, thus the essential factors that are to be targeted for AMD prevention, control and therapy development. To date, several attempts have been made to develop the specific drugs for the treatment of AMD, yet, there are no specific and effective drugs for the treatment of AMD^39^. Therefore, a new potential therapeutic drug is essential to be discovered for the better treatment of AMD.

Several lines of studies showed that cells upon exposure with H_2_O_2_ undergo various cellular stresses and finally get damaged. In the current study, we examined the anti-oxidant role of artemisinin upon oxidative stress mediated by ROS in RPE cells. In our *in-vitro* analysis, we found that D407 cells on exposure with 100 μM H_2_O_2_ for 24 h, about 30% of the cells underwent apoptotic cell death (Fig. 1 C); nonetheless very low concentration of artemisinin (10 μM) considerably reduced the cell death, changed the cellular morphology and decreased the LDH production in D407 cells induced by hydrogen peroxide as shown in Fig. 1, 2 and 3 respectively. These results are consistent and similar with our previous studies 31, 40 which suggested that artemisinin, commonly used as anti-malaria therapy also showed cytoprotective role against H_2_O_2_- toxicity via exerting its anti-oxidant property on to the cells.

Furthermore, RPE cells as described possesses abundant number of mitochondria and other cell organelles that produce intracellular ROS which in turn are associated with oxidative stress to cell. In fact, several reports showed that in AMD patients unexpectedly higher levels of intracellular ROS were observed in RPE cells along with damaged mitochondria that lead to the progression of AMD^16^, ^17^. Since, mitochondria and ROS both play a crucial role in AMD pathogenesis, ROS is targeted for AMD treatment and therapy. In our experiment, we designed an oxidative stress model by using H_2_O_2_ where H_2_O_2_ profoundly increased intracellular ROS in D407 cells and it consequently damaged mitochondria and decreased mitochondrial membrane potential (△ψm). Meanwhile, pre-treatment of cells with artemisinin substantially reduced the loss of mitochondrial membrane potential (△ψm) induced by H_2_O_2_ (Fig. 3B). In fact, pre-treatment with artemisinin might reduce the oxidative stress mediated by H_2_O_2_ and hence it protected the mitochondria from damage thereby preventing the further loss of mitochondrial membrane potential. Artemisinin here thus, might play anti-oxidant role in protecting the mitochondria and mitochondrial DNA from damage, which require further investigation.

Our previous studies have demonstrated that artemisinin protected human retinal pigment cell (D407), cortical neuron and PC12 cells against H_2_O_2_/SNP-induced cell apoptosis via activation of ERK/CREB and/or ERK/p38 MAPK signaling pathways respectively^30^, ^31^. The underlying mechanisms of protective role of artemisinin against H_2_O_2_-induced cell death is still not well clear. Some of previous reports have also shown that AMPK played a crucial role in cell protection^33^, ^34^, ^40^. Moreover, AMPK has also been found to be involved in sustaining cellular energy homeostasis, cancer cell survival and autophagy^41^, ^42^. However, the involvement of AMPK in artemisinin protection has not been previously described anywhere else. We thus, tried to disclose whether there is co-relation between AMPK and artemisinin protection. Indeed, we found the substantial activation of AMPK by both dose and time-dependent stimulation of human retinal pigmented (D407) cells with artemisinin (Fig. 4). Additionally, either in the presence of AMPK inhibitor, the compound C or knock-down of AMPK by si-RNA targeting AMPK, artemisinin failed to promote cell survival against H_2_O_2_ -induced cell death (Fig. 5). These results thus give a key mechanistic support suggesting that artemisinin promotes survival of human retinal pigment (D407) cells against H_2_O_2_ -induced cell death, at least in part, through modulation of AMPK. We have previously showed the regulation of ERK/CREB by artemisinin treatment. It was reported that AMPK phosphorylate CREB links our previous finding about ERK/CREB with our present observation about AMPK^43^. Interaction of ERK and AMPK was also reported in several studies^44^-^46^. These information put together pointed towards the possibility that AMPK can be an intermediary between artemisinin and activation of ERK/CREB. More importantly, similar results were also obtained in the human primary culture RPE cells. Nrf2 is an antioxidant transcription factor. AMPK acts as a central regulator of cell survival under stress-stimulated stimulation. It was reported that Nrf2 is the target of AMPK. Phosphorylation of Nrf2 by AMPK causes nuclear translocation of Nrf2 and increased transcription of Nrf2 target genes 47, 48. Nrf2 should be coordinated with the AMPK-controlled cell survival pathway, and it is possible that activation of AMPK by artemisinin increased the phosphorylation of Nrf2 which contributed to the survival protective/antioxidant effect of artemisinin. Consistent with this hypothesis, our preliminary results showed that AMPK phosphorylated Nrf2 in SH-SY5Y cells in another project. These results put together, indicated that artemisinin not only protected cell line of RPE from oxidative stress injury but also protected primary culture RPE cells via the activation of AMPK. Yet, further extensive study is required to explore the exact mechanism of artemisinin protection via AMPK activation.

**Figure 8:**
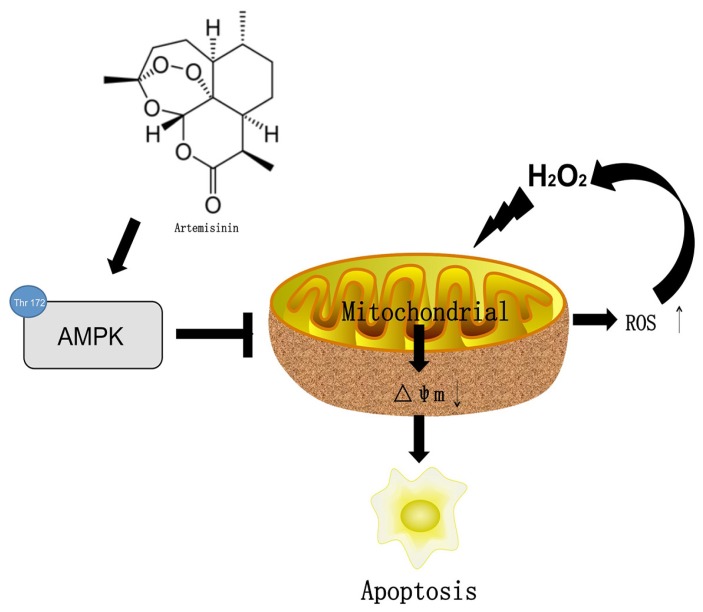
The mechamism of artemisinin

In conclusion, we herein demonstrated for the first time that artemisinin promotes RPE cell survival against H_2_O_2_ -mediated oxidative stress by activating the AMPK. Furthermore, we showed that artemisinin protection against H_2_O_2_ -induced cell damage involves various mechanistic routes including reducing intracellular ROS generation and inhibiting oxidative stress, decreasing LDH release and loss of mitochondrial membrane potential and changing the cell morphology (Fig. 8). Our current study thus, shows that artemisinin could be a potential therapeutic candidate for the treatment of AMD patients.

## Abbreviations

AMD: age-related macular degeneration
AMPK: AMP-activated protein kinase
H_2_O_2_: hydrogen peroxide
JC-1: 5,5′,6,6′-tetrachloro- 1,1′,3,3′-tetraethyl-benzimidazolyl-carbocyanineiodide
LDH: lactate dehydrogenase
ROS: reactive oxygen species
RPE: Retinal pigment epithelial
